# Supplementary material for: A Humanized Yeast Phenomic Model of Deoxycytidine Kinase to Predict Genetic Buffering of Nucleoside Analog Cytotoxicity
Source: Genes (Basel). 2019 Sep 30;10(10):770. doi: 10.3390/genes10100770 (PMC6826991; doi:10.3390/genes10100770)
Supplement: Supplementary file 1 [file genes-10-00770-s001.zip › Additional_Files/Additional_File7_GOTermHeatmaps.zip!/Additional_File7_GOTermHeatmaps/telomere_maintenance.pdf]

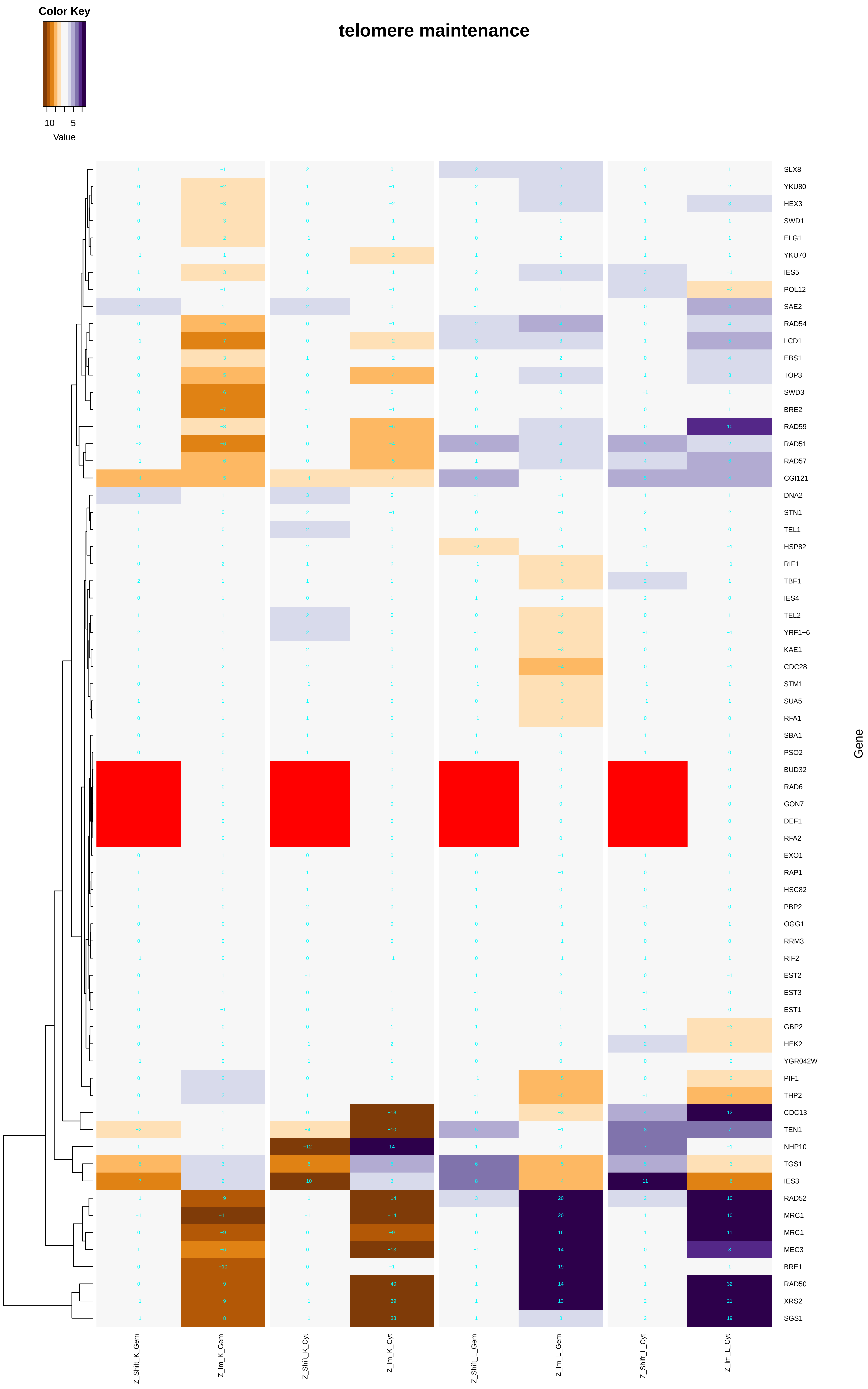
telomere maintenance
Color Key
−10
5
Value
SLX8
YKU80
HEX3
SWD1
ELG1
YKU70
IES5
POL12
SAE2
RAD54
LCD1
EBS1
TOP3
SWD3
BRE2
RAD59
RAD51
RAD57
CGI121
DNA2
STN1
TEL1
HSP82
RIF1
TBF1
IES4
TEL2
YRF1−6
KAE1
CDC28
STM1
SUA5
RFA1
SBA1
PSO2
BUD32
RAD6
GON7
DEF1
RFA2
EXO1
RAP1
HSC82
PBP2
OGG1
RRM3
RIF2
EST2
EST3
EST1
GBP2
HEK2
YGR042W
PIF1
THP2
CDC13
TEN1
NHP10
TGS1
IES3
RAD52
MRC1
MRC1
MEC3
BRE1
RAD50
XRS2
SGS1
Gene
Z_Shift_K_Gem
Z_Im_K_Gem
Z_Shift_K_Cyt
Z_Im_K_Cyt
Z_Shift_L_Gem
Z_Im_L_Gem
Z_Shift_L_Cyt
Z_Im_L_Cyt

telomere maintenance via recombination

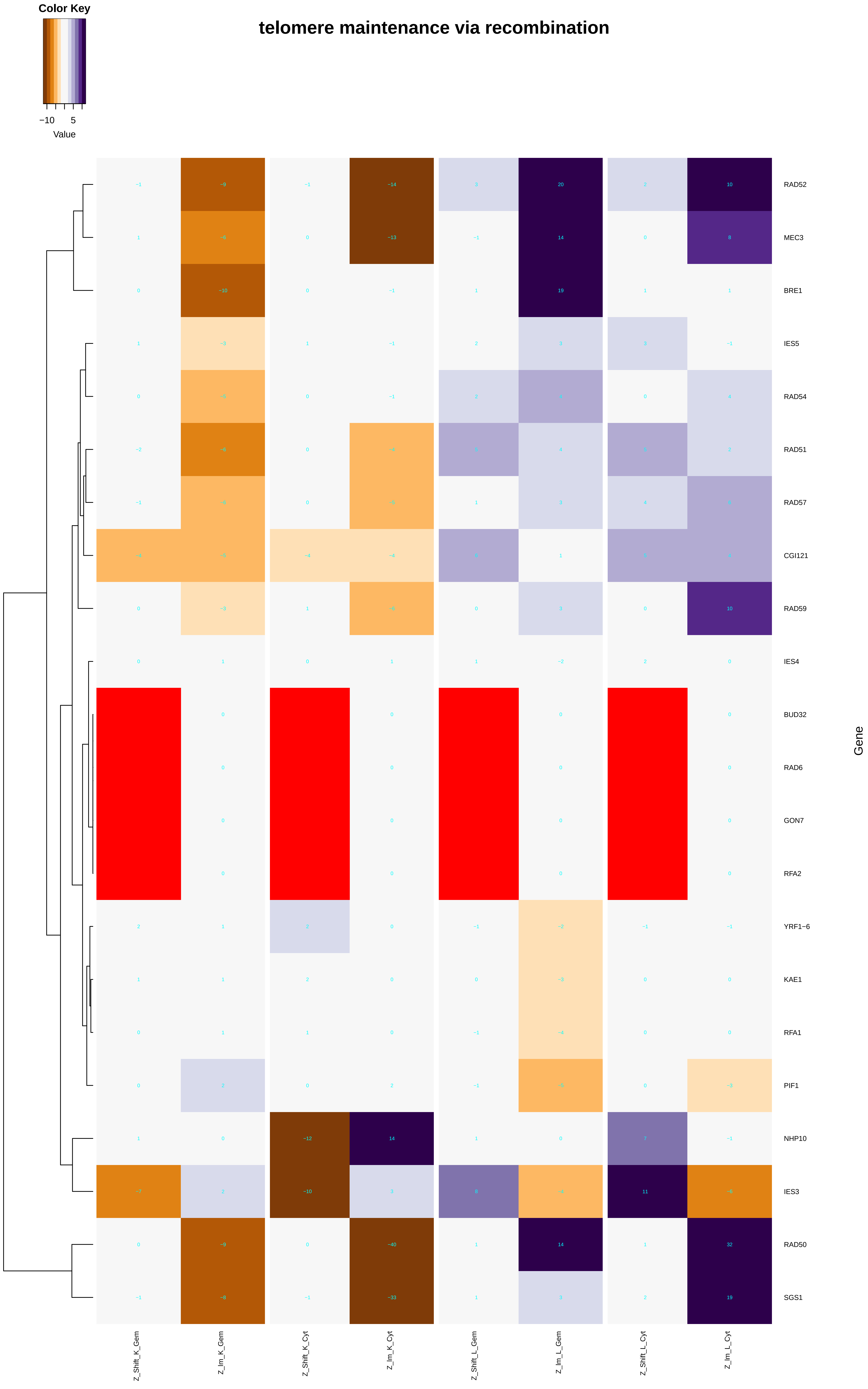

telomere maintenance via telomere lengthening

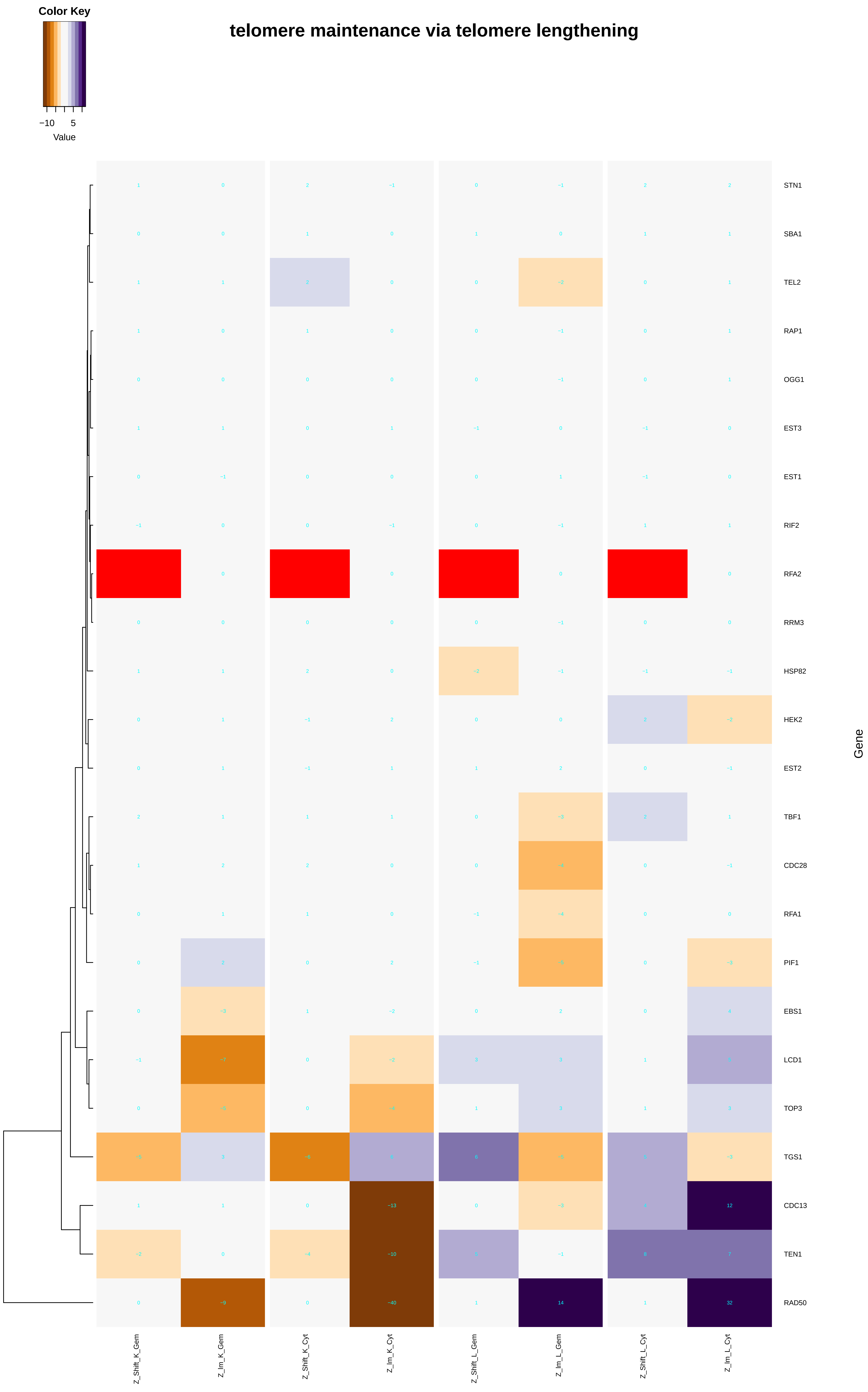

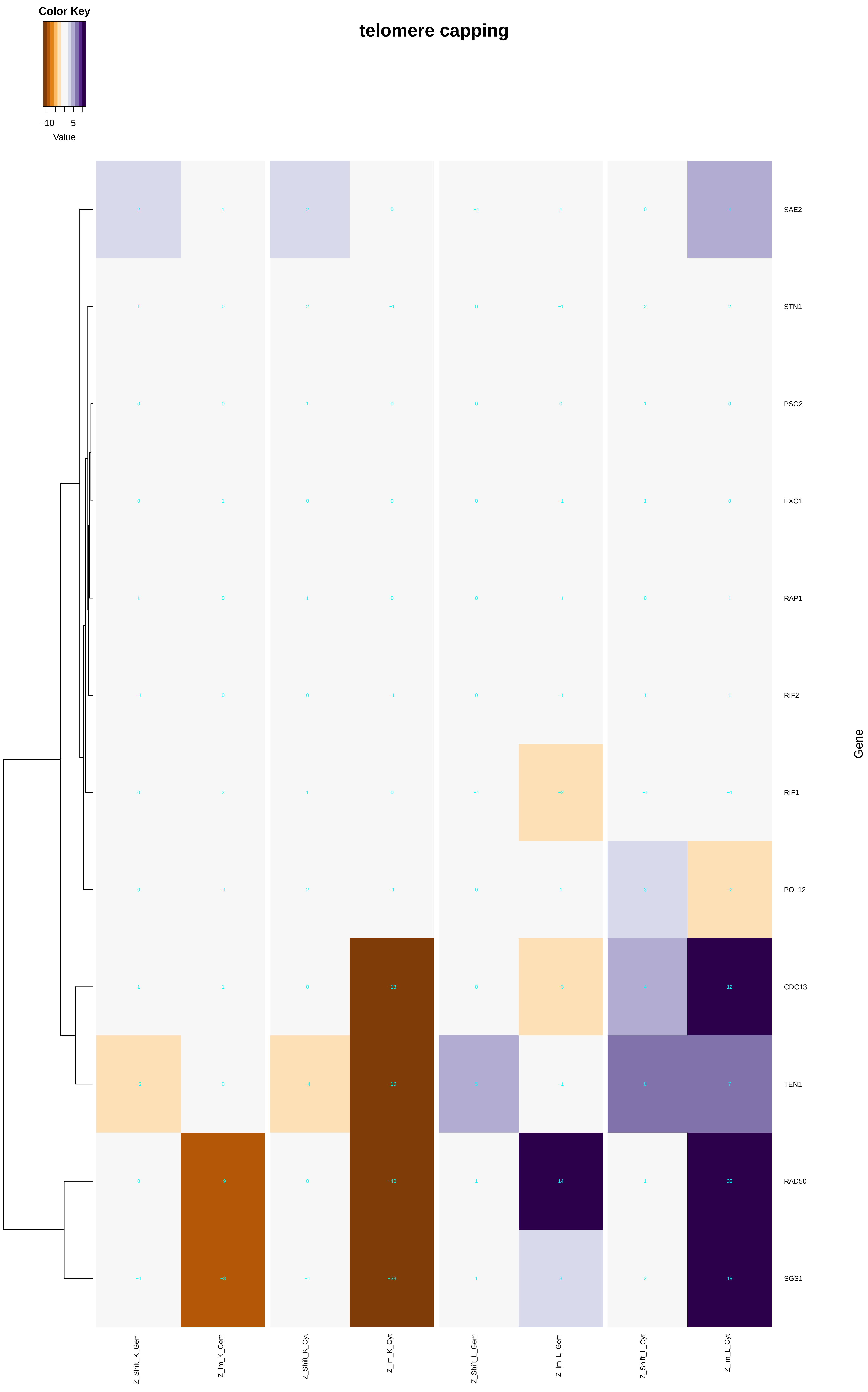
telomere capping
Color Key
−10
5
Value
SAE2
STN1
PSO2
EXO1
RAP1
RIF2
RIF1
POL12
CDC13
TEN1
RAD50
SGS1
Gene
Z_Shift_K_Gem
Z_Im_K_Gem
Z_Shift_K_Cyt
Z_Im_K_Cyt
Z_Shift_L_Gem
Z_Im_L_Gem
Z_Shift_L_Cyt
Z_Im_L_Cyt

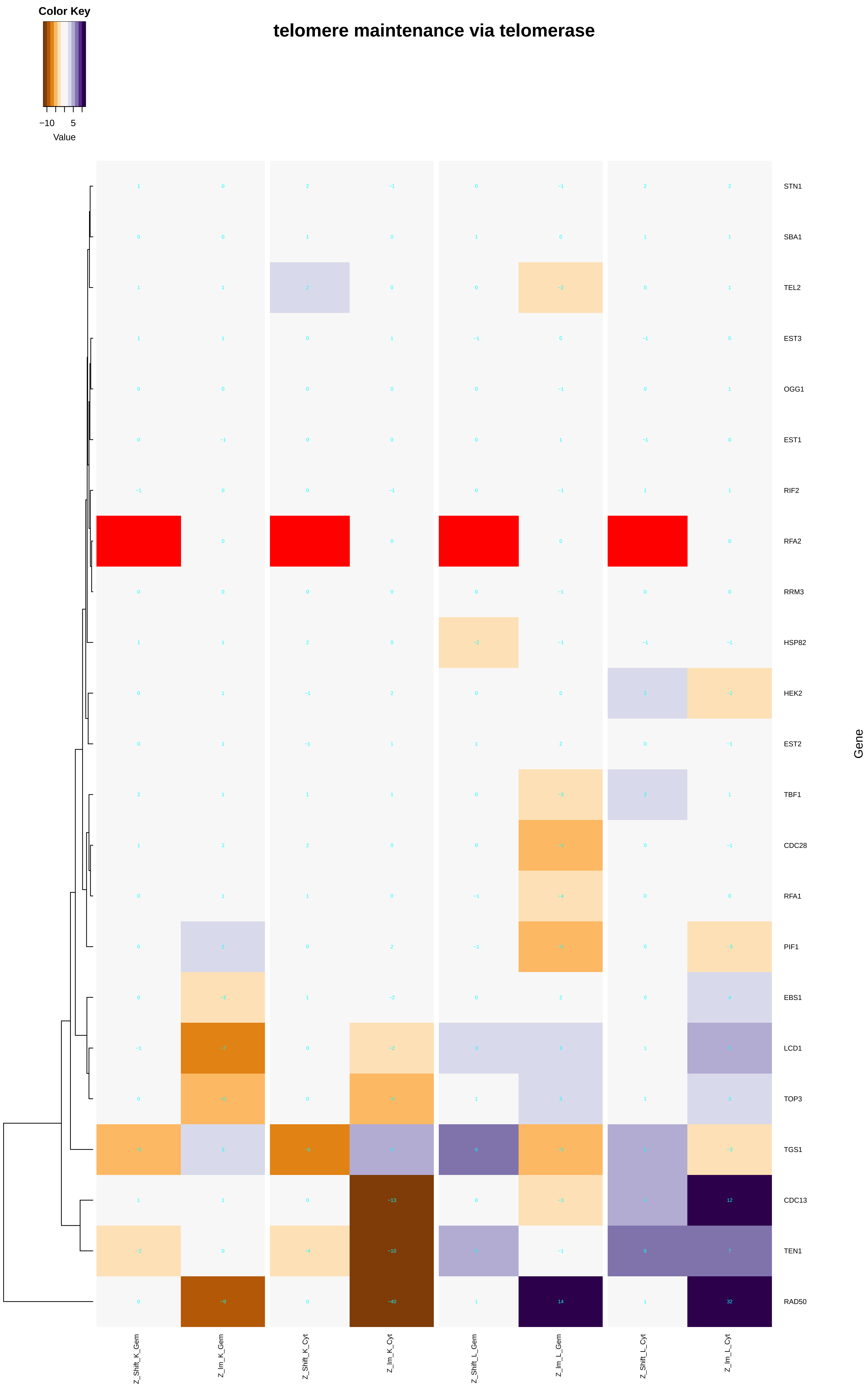
telomere maintenance via telomerase
Color Key
−10
5
Value
STN1
SBA1
TEL2
EST3
OGG1
EST1
RIF2
RFA2
RRM3
HSP82
HEK2
EST2
TBF1
CDC28
RFA1
PIF1
EBS1
LCD1
TOP3
TGS1
CDC13
TEN1
RAD50
Gene
Z_Shift_K_Gem
Z_Im_K_Gem
Z_Shift_K_Cyt
Z_Im_K_Cyt
Z_Shift_L_Gem
Z_Im_L_Gem
Z_Shift_L_Cyt
Z_Im_L_Cyt
